# Effect of Various Concentrations of Caffeine, Pentoxifylline, and Kallikrein on Hyperactivation of Frozen Bovine Semen

**DOI:** 10.1155/2015/948575

**Published:** 2015-04-09

**Authors:** Ibrahim A. H. Barakat, Mohamed A. Danfour, Fatma A. M. Galewan, Mohamed A. Dkhil

**Affiliations:** ^1^Zoology Department, College of Science, King Saud University, P.O. Box 2455, Riyadh 11451, Saudi Arabia; ^2^Cell Biology Department, National Research Centre, 33 Bohouth Street, Dokki, Giza 12622, Egypt; ^3^Physiology Department, Faculty of Medicine, Misurata University, Misurata, Libya; ^4^Zoology Department, Faculty of Science, Helwan University, Cairo 11795, Egypt

## Abstract

Caffeine, pentoxifylline, and kallikrein are substances that affect the efficiency of sperms in the fertilization process; however, they have not been adequately studied. The present study aimed to examine the influence of caffeine, kallikrein, and pentoxifylline on sperm motility in bovine as well as investigate their optimum concentrations for increasing the movement of sperms in bovine. Frozen bovine sperms were thawed in universal IVF medium supplemented with 1, 5, and 10 mM caffeine or pentoxifylline or 1, 4, and 8 U/mL kallikrein and were then incubated for 30 min. Treated semen parameters were analyzed using a computer assisted semen analyzer (CASA). Data analysis showed that the mean values concerning progression and motility of sperm increased in caffeine and pentoxifylline treatments when compared with the kallikrein group. The obtained results revealed that kallikrein is not necessary for the improvement of bovine sperm motility. Additionally, our results revealed that 5 mM from caffeine was the best concentration added to the medium, followed by 1 or 5 mM from pentoxifylline. Therefore, it is concluded from the present study that caffeine has hyperactivation efficacy at 5 mM concentration compared to other treatments.

## 1. Introduction

Assisted reproduction technologies (ART) were developed through the past few decades to produce high-yielding numbers of embryos. Comparable to other technologies, techniques of* in vitro* embryo production have their share of problems and failures [1], and, for that purpose, they need to be improved to produce viable embryos. Studies that investigated the materials having the ability to affect the integrity of sperm membranes, stimulate sperm motility, and suppress apoptosis and to improve fertilization ability of human and animal spermatozoa were correlated using the artificial insemination. Generally, artificial insemination programs widely use the diluted stored semen. The diluted and cold semen is used when the insemination is performed immediately after semen collection. When diluted and stored semen is to be used under farm conditions, the liquid semen may be easier [2]. Mammalian spermatozoa do not have the ability for fertilization immediately after ejaculation process; however, they gain this ability after passing the capacitation and hyperactivation process [3, 4]. Hyperactivity is one part of the continuous physiological processes occurring for sperm to acquire the fertilizing efficiency. Change in sperm motility gives the ability to penetrate the cumulus cells and zona pellucida surrounding the oocyte [4, 5]. Moreover, hyperactivation indicates a change in the sperm motility pattern from regular and symmetrical flagellate bends to high-amplitude and asymmetrical flagellate bends [6, 7]. Heparin, casein phosphopeptides, caffeine, pentoxifylline, and heparin plus caffeine were used for* in vitro* fertilization (IVF) process in order to decrease the embryonic development variation from different bulls [8–11].

Caffeine is an inhibitor for cyclic nucleotide phosphodiesterase resulting in an increase in the intracellular cyclic adenosine monophosphate (cAMP), stimulating capacitation and the spontaneous acrosome reaction of boar spermatozoa [12], and hence increasing sperm motility [13]. Also, caffeine may have a direct effect on cellular metabolism, and such effect depends on the concentration of calcium ions. Additionally, cattle IVF have been improved by the application of heparin alone or through its synergistic effects when used with caffeine [14].

The sperm motility was increased as a result of the conversion of kininogen into kinin by kallikrein which works as an enzyme [15]. Results in human [16] and bovine [17] showed the presence of kallikrein in seminal plasma. Kallikrein-kinin system, together with other factors, has a physiological role in mammal's semen, where it maintains and activates the motility of sperm. This assumption is based on investigations showing the stimulating effect of kallikrein and bradykinin on sperm motility of fresh bovine and ovine [18], as well as cryopreserved human [19] and human [20, 21] ejaculates. Moreover, the presence of kallikrein in the bull seminal plasma resulted in an increase of the sperm motility [22].

Pentoxifylline (PTX) works as a methylxanthine phosphor-diesterase inhibitor. It reduces superoxide anions and inhibits tumor-necrosis factor-alpha (TNF-alpha) responsible for DNA fragmentation and apoptosis or programmed cell death [23–25]. Additionally, it increases the intracellular cAMP [26], stimulates sperm motility, and improves the fertilization [10, 27]. Furthermore, Zhang et al. [28] showed that the application of PTX in clinical procedures leads to reduced lipid peroxidation associated sperm membrane damage and DNA apoptosis and scavenges the toxic reactive oxygen species. In laboratory and domestic species, many ingredients were used to study the hyperactivation of sperm, including kallikrein [15], thimerosal [29, 30], pentoxifylline (PTX) [10], procaine [31, 32], thapsigargin [29, 33], and caffeine [29].

Therefore, this study was an endeavor towards the improvement of semen quality by using caffeine, kallikrein, and pentoxifylline as sperm motility promoting factors.

## 2. Materials and Methods 

### 2.1. Chemicals and Media

Unless otherwise mentioned, all chemicals used in this study were purchased from Sigma Chemical Co. (St. Louis, MO, USA).

The medium used in this study was Universal IVF medium (MediCult, Origio company, Copenhagen, Denmark), which consisted of SSR (Synthetic Serum Replacement), HAS (Human Albumin Serum), glucose, sucrose, sodium lactate, physiological salts, glycerol, HEPES (4-(2-hydroxyethyl)-1-piperazineethane-sulfonic acid), sodium bicarbonate, penicillin, and streptomycin [34].

### 2.2. Experimental Design

In order to evaluate the effect of caffeine, pentoxifylline, and kallikrein on the activation of the bovine sperm, the thawed sperms were separately subjected to different concentrations from each of the tested materials in 10 tubes as shown in Table 1. After that, samples were divided into 10 equal sizes in 10 test tubes. The test tubes were incubated at 39°C for 30 min. After the incubation period, the tubes were investigated, and the percentages of movement as well as the speed of sperms treated with different concentrations of the tested compounds were measured to compare their effects on stimulating sperm movement. The tested concentration range was chosen according to previous studies performed with bovine sperm [29].

### 2.3. Freezing Bovine Semen

Thirty samples from bovine frozen semen (Holstein Strain, Friesian) were stored in liquid nitrogen at –96°C. Samples were imported from World Wide Sires Company, USA. Straws of frozen semen were thawed at 39°C for 20 sec and were then placed in sterile test tube containing high density medium [35]. In brief, one mL of the medium containing 80% density was placed in a test tube, and then 1 mL from the same medium containing 55% density was slowly added on the side of the tube to prevent mixing between the two layers of the media and to have a clear separation between them. Finally, one mL of the thawed bovine semen was added, and this tube was centrifuged at 700 g for 10 min. After centrifugation, the supernatant was discarded and the pellet was suspended with 1 mL IVF universal medium and was examined microscopically (100x) for motility. The tube containing the sperms was placed in 5% CO_2_ incubator at 39°C for 30 min until use.

### 2.4. Hyperactivation Test for Thawed Bovine Sperms

The most important measured traits were the movement and the hyperactivation of the sperms. Sperm hyperactivation can be assessed by analyzing specific motion parameters using CASA system. Hyperactivated and actual-path sperm tracing showed a star-shaped pattern [31, 32]. The motility parameters analyzed by the CASA system included (1) percentage of motile sperms (MOT%) or the percentage of moving sperms; (2) percentage of progressive sperm (PRO%) or the percent of sperms moving in a straight line path; and (3) percentage of nonmotile sperms (NMOT%) or the percent of sperms not moving forward.

### 2.5. Statistical Analysis

Data were analyzed by two-way repeated measurement ANOVA using the procedure of the Statistical Analysis System [36]. The results obtained were expressed as means ± standard error of mean (SEM). Significant differences between groups were obtained using Duncan test [37]. The differences between means were measured at *P* ≤ 0.05.

## 3. Results 

### 3.1. Influence of Caffeine, Pentoxifylline, and Kallikrein on Sperm Motivation

**Figure 1:**
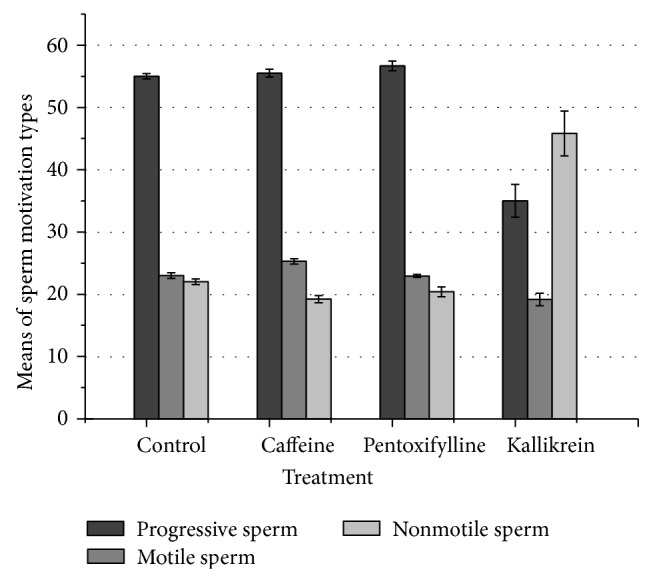
Effect of caffeine, pentoxifylline, and kallikrein on sperm motivation.

As shown in Figure 1, the mean for progressive sperms was highly significant in pentoxifylline treatment compared to the control and kallikrein treatments, while there is no significant difference between caffeine and pentoxifylline treatments. Concerning motile and nonmotile sperm traits, there were no significant differences between pentoxifylline versus control and pentoxifylline versus caffeine treatments, respectively. However, motile sperm trait showed significant differences between caffeine and kallikrein versus control and pentoxifylline treatments and between caffeine and kallikrein treatments. Additionally, the mean of nonmotile sperms was not significantly different between caffeine and pentoxifylline. In contrast, sperms treated with kallikrein showed a highly significant mean value for nonmotile sperms when compared with the control and other treatments.

### 3.2. Effect of Different Concentrations of Caffeine, Pentoxifylline, and Kallikrein on Sperm Motivation

**Figure 2:**
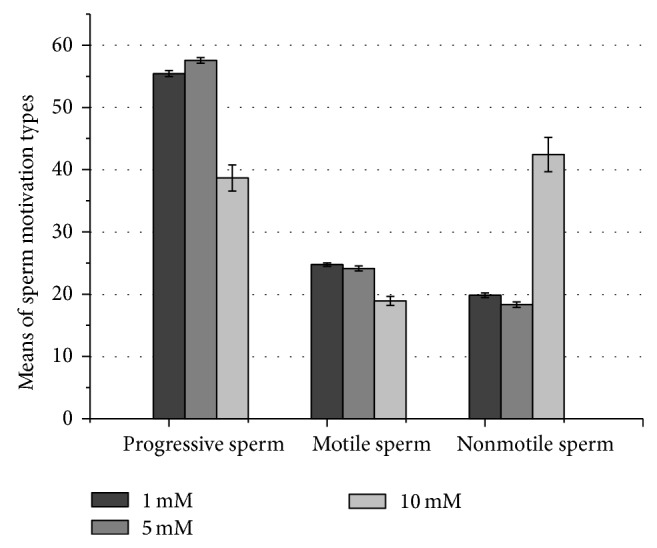
Effect of different concentrations of caffeine, pentoxifylline, and kallikrein on sperm motivation.

At first glance, for all compounds tested, both concentrations of 1 and 5 mM resulted in a significant increase in the progressive and motile sperm means when compared with the 10 mM concentration (Figure 2). On the other hand, the nonmotile sperm means were significantly lower at 1 and 5 mM when compared with 10 mM as shown in Figure 2.

### 3.3. Effect of Interaction between Different Concentrations and Compounds Tested on Sperm Motivation

Table 2 summarizes the analyzed data for this experiment. The results showed a significant increase in the progressive motile mean value (61.5 ± 0.8) with 5 mM caffeine, followed by 1 and 5 mM pentoxifylline (60.2 ± 1.2 and 60.0 ± 0.7, resp.). The zero mean value was obtained with 8 U/mL kallikrein. Additionally, it seems that the mean values for motile sperms were higher upon treating the sperms with 1 mM caffeine (26.2 ± 0.4) as well as 1 U/mL kallikrein (Table 2). Additionally, 8 U/mL kallikrein gave the highest significant mean value for nonmotile sperm trait, while the lowest significant mean value of 13.8 ± 0.4 was obtained with 5 mM caffeine. Analyzing the data in Table 2 showed that caffeine treatment was generally the best treatment giving significant increase in the progressive and motile sperm means and a decrease in the nonmotile sperm means. Moreover, kallikrein treatment resulted in a significant increase in the nonmotile sperm mean value (93.7 ± 0.4) at 8 U/mL, followed by 10 mM pentoxifylline (28.8 ± 0.8).

## 4. Discussion

Our study was carried out to investigate the effects of different concentrations of caffeine, pentoxifylline, and kallikrein on the movement of bovine frozen semen using a CASA system. The results showed that caffeine, a phosphodiesterase inhibitor, increased the bovine sperm motility depending on the concentration applied. The positive effects of caffeine and pentoxifylline on sperm motility were also demonstrated. The tested concentrations of caffeine and pentoxifylline improved the motility and progressive movement of sperms when added to the semen* in vitro*. However, the beneficial effect of caffeine and pentoxifylline was observed upon using 5 mM and 1 or 5 mM, respectively. It has been reported that intracellular calcium and immediate hyperactivation were increased by the addition of caffeine to ram sperm [38]. Previous studies reported that caffeine has many stimulating effects such as increasing sperm motility and inducing acrosome reaction and thus increasing the penetration rate and boar spermatozoa bound to the zona pellucida [2]. Moreover, sperms were able to penetrate the oocytes when they were capacitated in the fertilization medium without caffeine in porcine [39], pigs [40], and mice [41]. The total and progressive motility of bovine sperms were increased by using caffeine at 5 mM and incubation for 30 min as revealed in our study. On the other hand, using the caffeine at higher concentration may have adverse effects on the sperm. In rabbits, López and Alvariño [42] found that the sperm motility was increased at higher concentration (10 mM), whilst the lower concentrations (2.5 or 5 mM) did not affect sperm movement. In another study on human, a higher concentration of caffeine (>2.5 mM) showed adverse effects on sperm fertilization and the cleavage of embryos [43]. The same results were also reported in human [44] and bovine [45]. Therefore, Momozawa and Fuduka [46] recommended using the fertilization medium without caffeine in bovine IVF. However, caffeine has been shown to induce hyperactivation in bovine and cynomolgus macaque monkey sperm [29, 47], which is in agreement with our results. Interestingly, caffeine is believed to induce sperm hyperactivation by promoting the activation of Ca^2+^ permeable cation channels found in the plasma membrane [29]. The effect of caffeine on sperm characteristics may be species-specific; the sperm motility was adversely affected with high concentration of caffeine in ram [48, 49] and human at 5 mM; however, higher concentration in rabbit (10 mM) may increase the sperm motility [2].

Improvement of semen quality for storage and artificial insemination was achieved by enhancing the activity of sperms. Phosphodiesterase inhibitors such as pentoxifylline may result in a decrease in the cyclic adenosine monophosphate (cAMP) that plays a main role in sperm motility [50]. The beneficial effects of PTX in assisted reproductive technology are improving the sperm motility and fertilization ability in asthenozoospermia [51–53]. The beneficial effects of PTX in improving the bovine spermatozoa motility were studied in the present study. Although many previously reported studies showed that the sperm motility percentage was not significantly changed with PTX treatment [51, 54, 55, 57], however, our investigation gave different results, which were also in agreement with the work of McKinney et al. [57] performed on human sperm treated with PTX. Moreover, Brennan and Holden [58] demonstrated an enhancement of thawed sperm motility treated with PTX prior to cryopreservation. The superoxide in human spermatozoa was reduced by the addition of 5 mM PTX, where it works as a scavenger for oxygen-free radical [59]. Higher PTX concentrations, however, may be detrimental to membrane integrity [60]. Normal spermatozoa were protected from deleterious effects during cryopreservation through the addition of the 5 mM PTX [61]. Based on the previous results, PTX may be added to the cryomedium as a supplement. PTX may work as a cryoprotective agent in normal semen, where different concentrations are necessary to neutralize the excessive reactive oxygen species (ROS) as explained by Esteves et al. [62]. Our results showed that the treatment of bovine sperms with kallikrein led to a decrease in the progressive motility when compared with the caffeine and pentoxifylline treatment and control groups. In addition to the above findings, the mean value of sperm progression was zero upon treating the bovine semen with higher kallikrein concentration (8 U/mL) for 30 min. Due to the scarcity of previous studies dealing with the effect of kallikrein on sperm capacitation* in vitro*, therefore, the results of kallikrein effect are not intensively discussed. There is a relationship between the concentration of kallikrein in bovine seminal plasma and the motility of spermatozoa after ejaculation [22]. In another study, Somleva and Subev [17] confirmed this conclusion, which was also in agreement with Bratanov et al., Schill et al., and Leidl et al. [18, 21, 63] after the first investigation by Schill et al. [64] concerning the stimulating effect of kallikrein-kinin system components on sperm motility.

Collectively, our study has demonstrated that caffeine is the best material to increase the progression and motility of sperms in cattle and then pentoxifylline compared to kallikrein. Also, the study showed that lower concentrations are better than higher concentrations. Therefore, we recommend the addition of caffeine at 5 mM concentration to the fertilization medium.

## Figures and Tables

**Table 1:** Different conditions applied in studying the effect of caffeine, pentoxifylline, and kallikrein concentrations on the hyperactivation of bovine sperm.

Number	*In vitro* conditions	Treatment
1	Universal IVF medium	Control

2	Universal IVF medium + 1 mM	Caffeine
3	Universal IVF medium + 5 mM	
4	Universal IVF medium + 10 mM	

5	Universal IVF medium + 1 mM	Pentoxifylline
6	Universal IVF medium + 5 mM	
7	Universal IVF medium + 10 mM	

8	Universal IVF medium + 1 U/mL	Kallikrein
9	Universal IVF medium + 4 U/mL	
10	Universal IVF medium + 8 U/mL	

**Table 2:** Effect of different concentrations of caffeine, pentoxifylline, and kallikrein on the hyperactivation of frozen bovine spermatozoa.

Treatments	Concentrations	Progressive	Slowly	Dead
Control	0.0	55.0 ± 0.8^c^	23.0 ± 0.8^d^	22.0 ± 0.8^de^

Caffeine	1 mM	55.2 ± 0.7^c^	26.2 ± 0.4^a^	18.7 ± 0.4^f^
	5 mM	61.5 ± 0.8^a^	24.7 ± 1.1^bc^	13.8 ± 0.4^h^
	10 mM	49.8 ± 0.7^f^	25.0 ± 0.7^b^	25.2 ± 0.7^c^

Pentoxifylline	1 mM	60.2 ± 1.2^b^	23.7 ± 0.4^d^	16.2 ± 0.8^g^
	5 mM	60.0 ± 0.7^b^	23.8 ± 0.4^cd^	16.2 ± 0.8^g^
	10 mM	49.8 ± 1.2^f^	21.3 ± 0.4^e^	28.8 ± 0.8^b^

Kallikrein	1 U/mL	51.3 ± 0.4^e^	26.2 ± 0.4^a^	22.5 ± 0.5^d^
	4 U/mL	53.7 ± 0.4^d^	25.0 ± 0.7^b^	21.3 ± 0.4^e^
	8 U/mL	00.0 ± 0.0^g^	6.3 ± 0.4^f^	93.7 ± 0.4^a^

Mean values in the same columns with different superscripts (a, b, c, d, e, f, g, and h) differ significantly (*P* ≤ 0.05).
